# Talking About Sex: Sexual Communication in the Context of Sexual Revictimization

**DOI:** 10.1080/10538712.2026.2656300

**Published:** 2026-04-12

**Authors:** Erin E. Beckham, Mia S. Thompson, Jennifer C. Duckworth, Anna E. Jaffe

**Affiliations:** a University of Nebraska-Lincoln, Lincoln, NE, USA;; b University of Maryland Baltimore County, Baltimore, MD, USA;; c Washington State University, Pullman, WA, USA;; d University of Washington, Seattle, WA, USA

**Keywords:** Sexual assault, rape, childhood sexual assault, sexual communication, sexual health behaviors, safe sex behaviors, sexual satisfaction

## Abstract

The relationship between sexual victimization and communication is crucial to examine, given research suggesting that effective and frequent communication about sexual activities helps facilitate safer sex behaviors, sexual functioning, and pleasure. Building on prior studies in this area, which tend to focus on assertiveness, the current study examines childhood sexual abuse (CSA), adolescent/adulthood sexual assault (ASA), and communication frequency across three domains: safer sex behaviors, sexual interests, and consent/boundaries. The sample consisted of 422 sexually-active undergraduates recruited from four U.S. universities (M_age_ = 19.77; 76.5% women). Moderation analyses revealed that more severe ASA was associated with more frequent communication about safer sex and consent, but only for those with a history of CSA. The elevated frequency of communication following more severe and repeated sexual victimization may highlight survivors’ resilience in having ongoing conversations about safety in sexual situations.

## Sexual communication

Effective, high-quality, and frequent communication about sexual activities, including sexual preferences, desires, values, attitudes, and experiences, is critical to facilitating safer sex behaviors, sexual functioning, and sexual pleasure for both men and women (Harris et al., 2014; Snell et al., 1989). Communication about safer sex has been associated with increased condom use (Widman et al., 2014, 2022) and is recognized as a relevant component of interventions designed to reduce HIV risk (Gause et al., 2018). Communicating about one’s sexual needs and preferences is positively associated with sexual arousal, orgasm, male erectile function, less pain, and lubrication (Amidu et al., 2010; Kelly et al., 2006; Mallory et al., 2019). Additionally, discussing one’s sexual interests and desires has been theoretically (MacNeil & Byers, 2005) and empirically (Frederick et al., 2017; Montesi et al., 2011; Nuno, 2017) linked to sexual pleasure and satisfaction among men and women in relationships.

Despite the promise that sexual communication demonstrates in promoting safe and satisfying sexual encounters, many individuals face barriers to open dialogue about sex. For example, fear that sexual communication may threaten relationship stability, lead to shame and embarrassment, or result in one’s partner experiencing negative emotions may lead individuals to avoid sexual communication within their relationships (Rehman et al., 2019). Additionally, recent qualitative research on both men and women highlights that prior experiences of sexual coercion and the violation of sexual boundaries can serve as barriers to initiating sexual communication about consent in the future (Edwards et al., 2022). Given the numerous potential benefits of sexual communication, gaining a better understanding of it in the context of sexual (re)victimization – which has serious negative effects across multiple areas of functioning – may inform education and intervention strategies.

## Sexual victimization

Sexual victimization is characterized as any attempted or completed sexual act involving bodily contact (e.g., sexual touching, oral sex, penetrative sex) perpetrated without an individual’s freely given consent (e.g., using tactics such as force, threat of force, coercion; Basile et al., 2014; World Health Organization, 2024). Sexual victimization may include childhood sexual abuse (CSA; e.g., any sexual behavior inflicted on a child; Browne & Finkelhor, 1986) or sexual assault occurring during adolescence or adulthood (ASA). According to the National Intimate Partner and Sexual Violence Survey, 1 in 5 women in the U.S. experience attempted or completed rape at some point in their life and nearly one-quarter of men experience some form of sexual victimization (Smith et al., 2018).

Sexual victimization has been associated with a breadth of psychosocial problems including risky sexual behaviors (Arriola et al., 2005), suicide and non-suicidal self-injury (Dworkin et al., 2022; Maniglio, 2011), and psychological disorders (Amado et al., 2015; Dworkin, 2020; Hailes et al., 2019). Beyond the negative psychosocial sequelae faced by survivors of sexual victimization, many survivors are also subjected to revictimization later in life, often operationalized as experiencing both CSA and later ASA (Messman & Long, 1996). Indeed, survivors of sexual victimization are at increased risk for revictimization both across (Walker et al., 2019) and within developmental periods (Daigle et al., 2008; Humphrey & White, 2000; Jaffe et al., 2023). In fact, a recent meta-analysis identified prior victimization histories (sexual assault, partner violence) as the strongest correlates of experiencing sexual victimization in U.S. college students (Spencer et al., 2024). This pattern of revictimization is especially concerning, given that multiple sexual assaults can have a cumulative effect on psychosocial functioning. Survivors reporting multiple sexual victimizations have demonstrated higher levels of posttraumatic stress, anxiety, depression, and dissociation compared to survivors with fewer victimizations (Follette et al., 1996). Additionally, survivors who have experienced multiple instances of victimization frequently report more difficulties across multiple areas of functioning compared to survivors with fewer victimizations (Messman-Moore et al., 2000).

Sexual victimization can also have downstream impacts on interpersonal functioning, including in sexual situations (DiLillo, 2001). For example, Rothman et al. (2021) reported long-term impairments in emotional and sexual intimacy up to 9 years after experiencing a sexual assault in college. Indeed, ASA survivors have reported less frequent engagement in sexual activity (Georgia et al., 2018), as well as increased anxiety (Jozkowski & Sanders, 2012) and dissatisfaction (De Silva, 2001) during sexual encounters. Additionally, women who experienced CSA tend to report similar trends of dissatisfaction with relationships (Godbout et al., 2009) and sexual intimacy (Leonard & Follette, 2002). Further, prior work has found both CSA and ASA to be associated with difficulties with emotional intimacy among women, including distrust toward romantic partners (DiLillo & Long, 1999; Georgia et al., 2018). Moreover, research suggests that survivors of CSA and ASA are more susceptible to partner pressure to forgo condom use and engage in unprotected sex compared to their non-victimized peers (George et al., 2016). Despite a growing body of literature suggesting that sexual victimization negatively impacts the sexual and interpersonal functioning of survivors, research has seldom considered the relationship between CSA or ASA and sexual communication.

## Sexual victimization and sexual communication

A limited body of work examining sexual communication within the context of sexual victimization has largely focused on the related, yet distinct, construct of sexual assertiveness. For instance, sexual victimization may decrease survivors’ confidence in the utility of their assertiveness (Finkelhor, 1987) and lead to negative cognitions about the self and one’s worth (Dodd & Littleton, 2017). This difficulty with engaging in assertive communication may make it more difficult for survivors to assert their desires and needs during sexual encounters (Livingston et al., 2007). Consistent with this notion, a history of sexual victimization has been associated with lower assertiveness when refusing unwanted sexual advances (Morokoff et al., 1997) and less assertive communication around sexual preferences (Quina et al., 2000).

Although consent and sexual preferences are important components of sexual communication, prior research on sexual assertiveness does not fully capture the multifaceted nature of sexual communication. For instance, sexual communication includes the quantity and content of conversations around sex, as well as the frequency of communication (Mallory et al., 2019; Metts & Cupach, 1989). Frequency of sexual communication may be a particularly important metric for informing intervention efforts (Mallory, 2022) in that infrequent communicators may benefit from interventions targeting how to begin the conversation and communicate more frequently (Gause et al., 2018). Further, the frequency of sexual communication may vary across relevant topics (e.g., boundaries, preferences), and thus represent a more nuanced approach to understanding survivors’ sexual communication. For instance, it is unclear whether the impact of sexual victimization on communication is specific to discussions about sexual consent or whether it extends across a range of topics that limit safety and satisfaction in survivors’ sexual encounters.

Relatedly, prior work on communication has failed to consider victimization across developmental stages (i.e., revictimization), and rather has only examined CSA and ASA as separate constructs (Livingston et al., 2007; Quina et al., 2000). Consequently, the cumulative impact of revictimization on sexual communication is not well understood. Given the empirical basis for negative cumulative effects from repeated victimization (Follette et al., 1996; Messman-Moore et al., 2000), it is imperative to understand how sexual communication among survivors may differ by levels of victimization.

Finally, although an increasing number of studies have begun to consider male survivors (Anderson et al., 2018; Krahé & Berger, 2017), men remain underrepresented in the literature surrounding sexual victimization. From those studies that are inclusive of men, research highlights the importance of sexual communication for safer sex behaviors, sexual functioning, and sexual satisfaction (Amidu et al., 2010; Harris et al., 2014; Montesi et al., 2011; Widman et al., 2022). While there is comparatively less known about men’s sexual communication patterns, prior work indicates that there may be notable differences between men’s and women’s sexual communication. For instance, a study by Harris et al. (2014) found that sexual communication strategies, such as the directness of communication, may vary across biological sex. Additionally, prior research has identified gender differences in the relationship between sexual communication and relationship satisfaction (Montesi et al., 2011). Given the presence of notable gender and sex differences in general sexual communication, it is important to understand how these differences may be exacerbated within the context of sexual victimization.

## Current study

The current study seeks to examine how CSA and ASA relate to college students’ frequency of sexual communication, including communication about safer sex behaviors (e.g., birth control, condom use, testing for sexually transmitted infections), sexual interests, and sexual consent and boundaries. We address three aims within the current study. First, we examined the association between CSA history and the frequency of past-month communication about safer sex behaviors, sexual interests, and sexual consent. Given empirical support for impaired sexual intimacy and trust toward romantic partners following CSA (DiLillo & Long, 1999; Georgia et al., 2018; Leonard & Follette, 2002), we hypothesized that individuals with a history of CSA would engage in less frequent sexual communication compared to those without CSA. Second, we examined the association between ASA severity and frequency of past-month communication about safer sex behaviors, sexual interests, and sexual consent. Given the empirical associations between ASA and impaired sexual and relationship functioning (Rothman et al., 2021), as well as prior findings related to repeated instances of victimization (Follette et al., 1996; Messman-Moore et al., 2000), we hypothesized that individuals reporting a greater number of severe ASA experiences would engage in less frequent sexual communication compared to those reporting fewer, less severe ASA experiences. Third, we assessed for cumulative effects of revictimization – defined as experiences of both CSA and ASA – on the frequency of communication (i.e., whether the ASA-frequency association differed for those with CSA). Consistent with prior literature citing negative cumulative effects following revictimization, we hypothesized that individuals with a history of both CSA and ASA would have the lowest frequency of sexual communication compared to the rest of the sample.

## Methods

### Participants

The current study draws from a larger sample of participants enrolled in a multisite study examining the impact of the COVID-19 pandemic on college students’ mental health, drinking, and sexual experiences in 2021 (Jaffe et al., 2022, 2025). Of 1,016 total participants, 545 were excluded from current analyses because they did not endorse past-month sexual activity, 2 were excluded for not responding to the question on sexual communication, and 15 were excluded for not responding to questions on ASA severity. Given this study’s focus on the impact of victimization during emerging adulthood (i.e., ages 18–25; Arnett, 2000), an additional 16 participants were excluded for being above the age of 25. Finally, to ensure temporal precedence of our predictors and ensure that participants were not referring to an ASA experience when reporting on past-month sexual communication, 16 participants were excluded for reporting past-month ASA.

The final sample included 422 undergraduate students in the U.S. ages 18 to 25 who reported sexual activity in the past month from a university located in a mid-size Midwestern city (Site 1; *n* = 222), a large Southern city (Site 2; *n* = 85), a rural Northwestern town (Site 3; *n* = 68), and a large Northwestern city (Site 4; *n* = 47). The average participant was 19.77 years old (*SD* = 1.34). Participants were 76.5% cisgender women (*n* = 323), 21.8% cisgender men (*n* = 92), and 1.7% transgender and/or gender expansive (*n* = 7). Although most participants were exclusively heterosexual or straight (*n* = 309, 73.2%), over one-quarter of participants identified with a minoritized sexual identity (*n* = 113, 26.8%). Regarding race, most participants identified as White (*n* = 245, 58.1%), followed by Multiracial (*n* = 81, 19.2%), Black or African American (*n* = 45, 10.7%), Asian or Asian American (*n* = 43, 10.2%), or another race (*n* = 8, 1.9%). Finally, the majority (*n* = 272, 64.5%) of participants reported being in a committed relationship.

### Measures

#### Demographics

All participants were asked to provide self-report demographic information, including information on their age, race, gender, sexual orientation, recruitment site, and relationship status. Collection of sexual orientation and gender identity were informed by guidelines on best practices in clinical settings (Cahill & Makadon, 2014; Institute of Medicine, 2013).

#### Childhood sexual abuse

CSA history was assessed via a 3-item self-report screening measure, included as part of the larger Computer-Assisted Maltreatment Inventory – Child Sexual Abuse subscale (CAMI-CSA; DiLillo et al., 2010). The CAMI-CSA asked participants to indicate whether they have experienced intentional exposure to genitals or masturbation, sexual kissing, touching, or fondling, or attempted or completed intercourse (anal, oral, vaginal) prior to age 14. Participants responded “Yes” or “No” as to whether any of these sexual acts occurred against their will, with an immediate family member, or with someone more than 5 years older than them. The CAMI-CSA has demonstrated good psychometric properties (DiLillo et al., 2010). We considered someone as having CSA history (=1) if they responded “Yes” to any of the screening questions on the CAMI-CSA.

#### Adolescent/adulthood sexual assault severity

Experiences of ASA since age 14 were assessed via a version of the self-report Revised Sexual Experiences Survey (RSES; Koss et al., 2007) that was further modified to improve inclusivity of sexual and gender minority individuals (Canan et al., 2020). The RSES includes five types of unwanted sexual contact (e.g., touching, attempted or completed oral sex, attempted or completed penetration) and five tactics of perpetration (e.g., verbal pressure, anger or criticism, intoxication, threatening or physical harm, just doing the behavior without a chance to say no). For each contact-tactic combination, participants were asked to indicate the number of times (i.e., “*0*,” “*1*,” “*2*,” “*3–9*,” or “*10+*”) each event occurred since age 14. Those who reported one or more experiences of ASA were asked follow-up questions regarding the timing of the ASA event(s) with standard response options (e.g., “*Less than 1 month ago*”). To ensure severity scoring was consistent with prior work, we recoded responses to reflect a narrower range of possible occurrences per item (i.e., “*0*,” “*1*,” “*2*,” or “*3+*”), then computed severity scores on a 0–63 scale (Davis et al., 2014). Lower severity scores represented fewer instances of unwanted touching, and greater severity scores represented more instances of completed rape.

#### Sexual communication

Participants were asked to indicate how frequently they engaged in each domain of communication with their sexual partner(s) over the past month. They were asked to respond to self-report prompts on a 0 (“*Never*”) to 4 (“*Very often*”) Likert-type scale to indicate the frequency of communication around safer sex behaviors (e.g., birth control use, condom use), sexual interests, and sexual consent or boundaries.

### Procedure

All study procedures were approved by each site’s Institutional Review Board. Undergraduate students at each of the four sites were offered participation in the larger study based on enrollment in psychology or human development courses. At three sites, participants were recruited using the undergraduate psychology department participant pool; these students were awarded research credits for participation, which count toward class participation. At the final site, students were able to complete research, including the current study, for extra course credit in a human development class. Informed consent and cross-sectional questionnaire data were recorded electronically through Qualtrics.

### Analytic plan

Analyses were conducted using R version 4.4.3 (R Core Team, 2025) and included the following packages: *tidyverse* (Wickham et al., 2019), *psych* (Revelle & Revelle, 2015), *descr* (Aquino et al., 2021), *gtsummary* (Sjoberg et al., 2021), *sjPlot* (Lüdecke, 2021), and *sjmisc* (Lüdecke, 2018). We first ran descriptive statistics and correlations for all study variables. Next, to examine the impact of CSA, ASA, and revictimization on communication we examined main effect models and then conducted moderation analyses testing whether CSA moderates the association between ASA severity and each domain of communication. Covariates for all analyses included age, relationship status, sexual minority status, gender, and recruitment site. Categorical covariates were dummy coded, including sexual minority status, gender (i.e., cisgender woman, cisgender man, transgender and/or gender expansive), and recruitment site (i.e., Sites 1–4). The largest group was selected to serve as the reference group for sexual minority status (i.e., heterosexual), gender (i.e., cisgender woman), and site (i.e., Site 1). Conditional significant interaction effects were probed by examining simple slopes.

## Results

### Descriptive statistics

One-fifth of participants endorsed a history of CSA (*n* = 84, 19.9%), and nearly one-half of participants endorsed experiencing ASA (*n* = 183, 43.4%). On average, participants endorsed relatively low levels of ASA severity (*M* = 7.91, SD = 13.75), although severity scores spanned the entire 0–63 range of severity. Individuals with a history of CSA tended to endorse significantly higher ASA severity compared to individuals without a history of CSA, *t*(97.31) = −5.18, *p* < .001.

See Table 1 for descriptive statistics and bivariate correlations between study variables. On average, participants endorsed “*Sometimes*” communicating about each sexual domain, with frequencies spanning the full 0–4 range of frequency for each domain. Across domains of sexual communication, frequency was related but not redundant (*r*s = .45 to .67), suggesting that individuals frequently engaging in one domain of sexual communication were also frequently communicating about other domains.

### Main effects between victimization and communication

Regressions examined the unique associations between CSA and ASA severity on the frequency of sexual communication across three domains (see Table 2). There were no significant main effects between CSA and the communication domains of safer sex behaviors (*B* = −0.20, *p* = .231), sexual interests (*B* = 0.16, *p* = .292), and sexual consent or boundaries (*B* = −0.12, *p* = .481). Additionally, no significant main effects were found between ASA severity and the three domains of communication (safer sex behaviors: *B* = 0.00, *p* = .615; sexual interests: *B* = 0.00, *p* = .567; sexual consent or boundaries: *B* = 0.01, *p* = .258). As indicated by *R*^*2*^, model predictors accounted for 10.4% of the variance in communication about safer sex behaviors, 3.6% of the variance in communication about sexual interests, and 2.7% of the variance in communication about sexual consent or boundaries.

### CSA as a moderator of the association between ASA & sexual communication

Regressions evaluated whether CSA moderated the association between ASA severity and frequency of communication around safer sex behaviors, sexual interests, and consent/boundaries respectively, while controlling for covariates. Full results are shown in Table 3.

**Figure 1.**
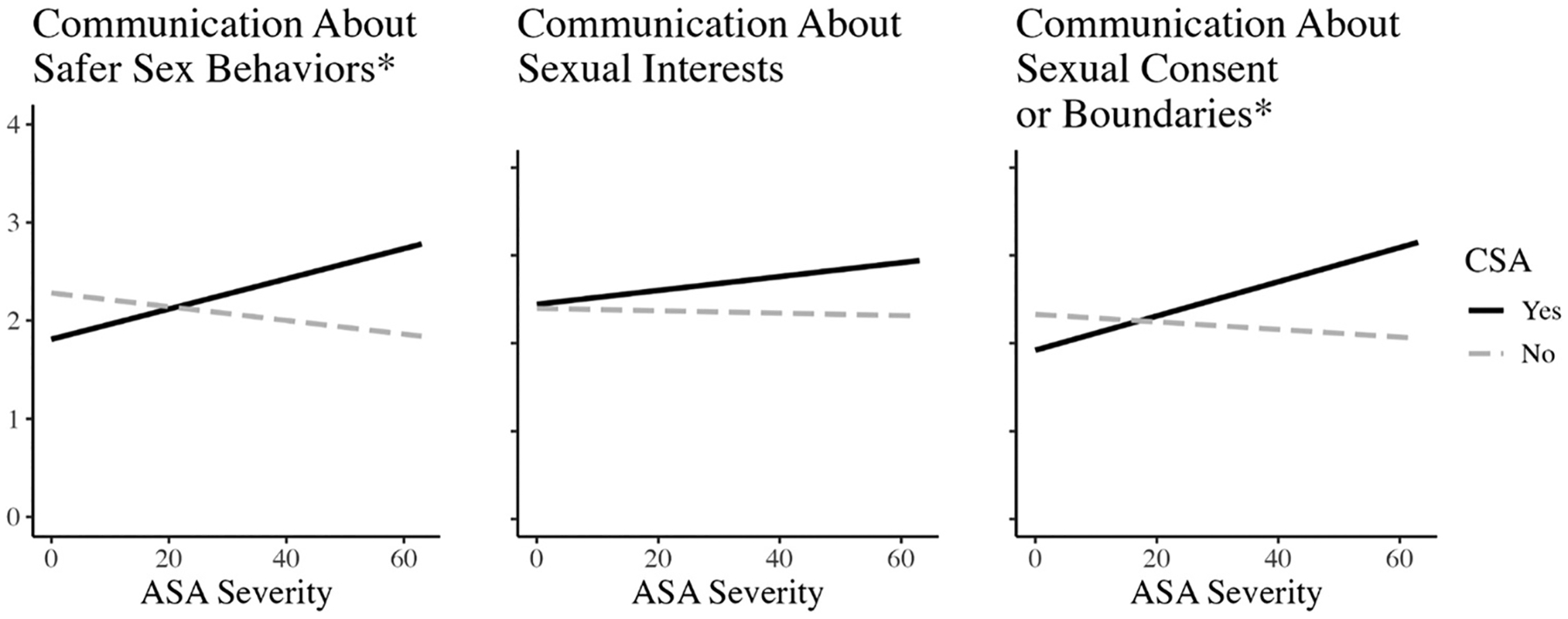
Simple slopes. *Note*. *Interaction significant at *p* < .05. CSA = childhood sexual abuse. ASA = adolescent/adulthood sexual assault.

There was a significant interaction between CSA history and ASA severity in the prediction of safer sex communication (*B* = 0.02, *p* = .020), indicating that the association between ASA severity and frequency of safer sex communication significantly differed based on history of CSA (Figure 1). Simple slope analyses revealed that for those with a history of CSA, more severe ASA was associated with more communication around safer sex behaviors (*B* = 0.02, *p* = .04). For those without a history of CSA, ASA severity was not associated with communication about safer sex behaviors (*B* = −0.01, *p* = .28). Further, when ASA severity was held to zero (i.e., no ASA), CSA was associated with less frequent communication about safer sex (*B* = −0.47, *p* = .021). As indicated by *R*^*2*^, 11.6% of the variance in safer sex communication was accounted for by model predictors.

Communication regarding sexual interests was not significantly predicted by CSA history (*B* = 0.05, *p* = .799), ASA severity (*B* = −0.00, *p* = .816), nor their interaction (*B* = 0.01, *p* = .287), revealing that CSA did not moderate the relationship between ASA severity and sexual interest communication within this sample. As indicated by *R*^*2*^, 3.8% of the variance in communication about sexual interests was accounted for by model predictors.

Finally, there was a significant interaction between CSA history and ASA severity in the prediction of consent/boundary communication (*B* = 0.02, *p* = .016), indicating that the association between ASA severity and frequency of consent/boundary communication differed by history of CSA. Simple slope analyses revealed that for those with a history of CSA, more severe ASA was associated with more communication around sexual consent or boundaries (*B* = 0.02, *p* = .01). For those without a history of CSA, ASA severity was not associated with communication about consent/boundaries (*B* = 0.00, *p* = .51). Further, when ASA severity was held to be 0, CSA history was associated with less frequent communication about consent and boundaries (*B* = −0.41, *p* = .049). As indicated by *R*^*2*^, 4.1% of the variance in communication about sexual consent or boundaries was accounted for by model predictors.

## Discussion

This study expands existing research on sexual assertiveness following sexual victimization by examining the cumulative effects of CSA and ASA on three domains of sexual communication: safer sex behaviors, sexual interests, and navigating sexual consent and boundaries. In this large, mixed-gender sample of college students, the frequency of sexual communication across three domains was interrelated but distinct, and differentially related to one’s personal history of sexual victimization and revictimization. Although no main effects were found between either CSA or ASA and any domain of communication, we found several notable interaction effects. As detailed below, sexual victimization history was most strongly linked to frequency of sexual communication about safer sex behaviors (e.g., condom negotiation) and sexual consent or boundaries, and not related to frequency of communication about sexual interests.

First, we assessed whether those with a CSA history would report engaging in less frequent sexual communication than those without a CSA history across domains. Although no main effects of CSA were found, simple effects of CSA were observed for participants *without* an ASA history, perhaps because the impact of CSA on communication could be more precisely examined in this subgroup. Specifically, those with a history of CSA (but no ASA) reported significantly less frequent communication about safer sex behaviors and consent and boundaries than those without a history of CSA or ASA, consistent with our predictions and prior literature (Livingston et al., 2007). Similarly, sexual victimization has been associated with less sexual assertiveness (Morokoff et al., 1997), less condom negotiation, greater abdication in sexual interactions (e.g., Braham et al., 2019; George et al., 2016), and ultimately, increased likelihood of unprotected sex (Masters et al., 2014), and revictimization (Jaffe et al., 2023). Reduced frequency in safer sex and boundary-setting communication observed in those with CSA histories in this sample may contribute to these health risk behaviors. Of note, the link between CSA history and sexual communication was only observed for the domains of safer sex behaviors and sexual consent and boundaries, with no significant differences for sexual interests communication. Although findings merit replication in larger samples, this distinction may reflect negative impacts of CSA history on survivors’ ability to protect against physical health risks in sexual interactions.

Second, we assessed ASA severity as a predictor of sexual communication frequency. Although again, no significant main effect of ASA severity was observed, simple effects can be interpreted. For participants without a CSA history, ASA severity was not significantly associated with any of the three communication domains. This lack of significance contrasts prior literature suggesting that survivors may have difficulty with assertive communication related to their sexual desires and needs (Livingston et al., 2007). This finding may reflect the strength of cumulative negative effects following repeated victimization (Follette et al., 1996; Messman-Moore et al., 2000); it is possible that neither CSA nor ASA severity alone is a unique predictor of sexual communication frequency.

Third, when examining the cumulative effects of CSA and ASA, current findings revealed significant interaction effects of sexual victimization across developmental periods (i.e., CSA to ASA revictimization) on sexual communication surrounding safer sex behaviors and consent/boundaries, though not in the direction we expected, nor consistent with prior findings. Contrary to our hypothesis, those with a history of both CSA and severe ASA reported the *most frequent* communication, and this finding was consistently demonstrated for the domains of communication about safe sex and consent/boundaries. One possible explanation is that those who have been impacted by repeated and severe sexual violence may be most motivated to regularly discuss sexual safety with their partners. Indeed, research indicates that some survivors have reported practicing increased assertiveness during sexual encounters to reduce their risk of revictimization (Ullman et al., 2018). Notably, these associations did not extend to communication about sexual interests, potentially suggesting that survivors are primarily focused on protecting against risks during sexual interactions instead of enhancing their sexual satisfaction. Given that survivors can also experience decreased interest in sex after an assault (O’Callaghan et. al., 2019), communicating about sexual safety may take precedence over sexual interests and pleasure. Overall, our unexpected findings may highlight survivors’ resilience in making frequent attempts to communicate about safety and boundaries with their sexual partners.

Our findings provide important insight into how a history of CSA and ASA severity can be associated with different types of sexual communication within survivors’ future sexual encounters. Results indicate that examining CSA and ASA severity together may offer a nuanced view of the unique and cumulative effects of prior sexual victimization on survivors’ subsequent sexual communication.

### Limitations and future directions

The current study is not without limitations. First, past-month frequency of communication was assessed retrospectively, leaving room for error and recall bias. However, the short time frame (i.e., past-month) likely decreased participants’ recall bias. Future studies implementing daily diary or ecological momentary assessment could facilitate more accurate reporting of sexual communication. Secondly, the current analyses relied on calculated ASA severity scores. Although these scoring decisions have been comprehensively compared to alternatives (Davis et al., 2014), they nonetheless involve assumptions about the relative severity of different perpetrator tactics and may lead to different findings than subjective severity reports about the impact of events from survivors themselves. Finally, data were collected during the COVID-19 pandemic; restrictions and safety protocols may have led to differences in sexual encounters and sexual communication that could complicate the generalizability of results.

Future research should examine the various contextual and situational factors that may impact the outcomes of survivors’ sexual communication, even in those instances when they are communicating frequently. For example, although survivors may talk with their partners about boundaries regularly, if they have difficulties with assertiveness, these discussions may not lead to the desired outcomes. Further, frequent communication may reflect more significant difficulties with sexual functioning. In contrast, survivors could utilize perfect communication skills with a non-receptive partner unwilling to act upon these discussions, and still not accomplish their communication goals. Taken together, it may be important for future studies to distinguish survivors’ communication attempts from their communication effectiveness. Moreover, it may be valuable to examine the combined effects of sexual communication frequency and effectiveness on survivors’ sexual functioning. Future research should also evaluate survivors’ motivations for sexual communication (e.g., risk mitigation, enhancement of sexual satisfaction), as these may provide clarity to the current results. Moreover, expanding the focus of sexual communication research toward understanding resilience, rather than focusing solely on negative sequelae following sexual victimization and revictimization, would likely be beneficial for survivor education and intervention efforts.

### Clinical implications

Although more research is needed to elucidate underlying mechanisms, the current results have several implications for clinical practitioners working with sexual assault survivors. First, since individuals experiencing CSA (without ASA) reported less frequent communication about safer sex behaviors than those with no sexual victimization histories, psychoeducation on assertive communication within sexual relationships following CSA disclosure may be indicated. Given concerns about the potential negative reactions that survivors’ sexual partners may have to boundary setting, practitioners should consider incorporating skill-building practices to enhance survivor self-efficacy in sexual communication within a supportive context. Finally, as results indicated that survivors tended to discuss their sexual interests less frequently than other domains of communication, practitioners could focus on helping survivors explore their definitions of sexual pleasure. Survivors who have experienced revictimization might benefit from the use of motivational interviewing techniques that emphasize their success in other domains of sexual communication to encourage discussion of their sexual interests more frequently, to the extent that this aligns with their goals.

## Conclusion

The present study investigated the association between CSA history, ASA severity, and revictimization on past-month sexual communication frequency across three domains. Unexpectedly, only communication about safer sex behaviors and sexual consent and boundaries were associated with CSA history, and ASA severity was not associated with any of the communication domains in isolation. However, results suggested a cumulative effect of CSA history and severe ASA, such that those who experienced revictimization reported more frequent communication about safer sex behaviors and consent and boundaries, but not sexual interests. Findings suggest that more severe and repeated sexual victimization experiences may motivate more frequent communication to prevent negative sexual outcomes, including nonconsensual sex, unwanted pregnancy, and sexually transmitted infections. Although survivors of sexual victimization tend to experience more difficulties with sexual satisfaction and functioning (e.g., De Silva, 2001; DiLillo, 2001), current findings suggest this may not translate into more frequent communication about sexual interests. More research is needed to understand how to leverage survivors’ frequent conversations about preventing negative outcomes to also help them reengage in satisfying and pleasurable sexual encounters.

## Figures and Tables

**Table 1.** Descriptive statistics and correlations between study variables.

			Correlations						
Variable	*M* (*SD*) / *n* (%)	Observed Range	1	2	3	4	5	6	7
1. Sexual Minority	113 (26.8%)	0, 1							
2. Age	19.77 (1.34)	18 – 24	.04						
3. Relationship	272 (64.5%)	0, 1	.04	.11*					
4. CSA history	84 (19.9%)	0, 1	.23**	.03	.07				
5. ASA severity	7.91 (13.75)	0 – 63	.26**	.04	.00	.33**			
Communication frequency regarding …									
6. Safer Sex Behaviors	2.04 (1.32)	0 – 4	−.03	−.16**	−.05	−.07	.00		
7. Sexual Interests	2.37 (1.14)	0 – 4	.10*	−.04	.12**	.08	.06	.45**	
8. Consent	2.27 (1.30)	0 – 4	.06	−.05	.03	−.02	.03	.56**	.67**

*Note*. CSA = childhood sexual abuse, ASA = adolescent/adulthood sexual assault.

* *p* < .05.

** *p* < .01.

**Table 2.** Main effects models predicting sexual communication frequency.

	Safer Sex Behaviors			Sexual Interests			Sexual Consent or Boundaries		
Predictor	*B*	*SE*	*p*	*B*	*SE*	*p*	*B*	*SE*	*p*
Site 2 (vs. 1)	0.06	0.17	.728	−011	0.15	.464	−0.09	0.17	.614
Site 3 (vs. 1)	−0.58	0.18	.001**	−0.24	0.16	.139	−0.36	0.18	.047*
Site 4 (vs. 1)	0.42	0.21	.044*	0.01	0.18	.956	0.18	0.21	.387
Sexual minority (vs. heterosexual)	−0.06	0.15	.684	0.22	0.13	.103	0.15	0.15	.316
Age	−0.17	0.05	<.001***	−0.06	0.04	.178	−0.06	0.05	.219
In a committed relationship	−0.11	0.13	.379	0.28	0.12	.018*	0.10	0.13	.467
Cisgender man (vs. cisgender woman)	−0.49	0.15	.002**	0.05	0.14	.698	0.18	0.16	.248
TGE (vs. cisgender woman)	−1.10	0.49	.027*	0.02	0.44	.972	0.44	0.50	.388
CSA history	−0.20	0.17	.231	0.16	0.15	.292	−0.12	0.17	.481
ASA severity	0.00	0.00	.615	0.00	0.00	.567	0.01	0.01	.258

Note.

* *p* < .05.

** *p* < .01.

*** *p* < .001. Site 1 = university in a mid-size Midwestern city. Site 2 = university in a large Southern city. Site 3 = university in a rural Northwestern town. Site 4 = university in a large Northwestern city. TGE = transgender/gender expansive. CSA = childhood sexual abuse. ASA = adolescent/adulthood sexual assault.

**Table 3.** Interaction models predicting sexual communication frequency.

	Safer Sex Behaviors			Sexual Interests			Sexual Consent or Boundaries		
Predictor	*B*	*SE*	*p*	*B*	*SE*	*p*	*B*	*SE*	*p*
Site 2 (vs. 1)	0.07	0.17	.690	−0.11	0.15	.478	−0.08	0.17	.648
Site 3 (vs. 1)	−0.59	0.18	.001**	−0.24	0.16	.130	−0.38	0.18	.038*
Site 4 (vs. 1)	0.41	0.20	.044*	0.01	0.18	.960	0.18	0.21	.390
Sexual minority (vs. heterosexual)	−0.07	0.15	.622	0.21	0.13	.112	0.14	0.15	.358
Age	−0.17	0.05	<.001***	−0.06	0.04	.178	−0.06	0.05	.216
In a committed relationship	−0.11	0.13	.382	0.28	0.12	.018*	0.10	0.13	.457
Cisgender man (vs. cisgender woman)	−0.52	0.15	.001***	0.04	0.14	.773	0.15	0.16	.349
TGE (vs. cisgender woman)	−1.14	0.49	.021*	−0.00	0.44	.999	0.39	0.50	.433
CSA history	−0.47	0.20	.021*	0.05	0.18	.799	−0.41	0.21	.049*
ASA severity	−0.01	0.01	.277	−0.00	0.01	.816	−0.00	0.01	.511
CSA history X ASA severity	0.02	0.01	.020*	0.01	0.01	.287	0.02	0.01	.016*

Note.

* *p* < .05.

** *p* < .01.

*** *p* < .001. Site 1 = university in a mid-size Midwestern city. Site 2 = university in a large Southern city. Site 3 = university in a rural Northwestern town. Site 4 = university in a large Northwestern city. TGE = transgender/gender expansive. CSA = childhood sexual abuse. ASA = adolescent/adulthood sexual assault.

## Data Availability

Data are not publicly available. Requests can be made to the corresponding author.
